# Structural Insights into the Ligand–LsrK Kinase Binding Mode: A Step Forward in the Discovery of Novel Antimicrobial Agents

**DOI:** 10.3390/molecules28062542

**Published:** 2023-03-10

**Authors:** Roberta Listro, Giorgio Milli, Angelica Pellegrini, Chiara Motta, Valeria Cavalloro, Emanuela Martino, Johannes Kirchmair, Giampiero Pietrocola, Daniela Rossi, Pasquale Linciano, Simona Collina

**Affiliations:** 1 Department of Drug Sciences, University of Pavia, Viale Taramelli 12, 27100 Pavia, Italy; 2 Department of Molecular Medicine, Biochemistry Unit, University of Pavia, Viale Taramelli 3/b, 27100 Pavia, Italy; 3 Department of Earth and Environmental Sciences, University of Pavia, Via Sant ’Epifanio 14, 27100 Pavia, Italy; 4 Division of Pharmaceutical Chemistry, Department of Pharmaceutical Sciences, University of Vienna, Josef-Holaubek-Platz 2, 2D 303, 1090 Vienna, Austria

**Keywords:** LsrK, antimicrobial resistance, tryptophan fluorescence spectroscopy, circular dichroism, docking, screening, LsrK binders, biofilm inhibitors

## Abstract

LsrK is a bacterial kinase that triggers the quorum sensing, and it represents a druggable target for the identification of new agents for fighting antimicrobial resistance. Herein, we exploited tryptophan fluorescence spectroscopy (TFS) as a suitable technique for the identification of potential LsrK ligands from an in-house library of chemicals comprising synthetic compounds as well as secondary metabolites. Three secondary metabolites (**Hib-ester**, **Hib-carbaldehyde** and **(*R*)-ASME**) showed effective binding to LsrK, with KD values in the sub-micromolar range. The conformational changes were confirmed via circular dichroism and molecular docking results further validated the findings and displayed the specific mode of interaction. The activity of the identified compounds on the biofilm formation by some *Staphylococcus* spp. was investigated. **Hib-carbaldehyde** and **(*R*)-ASME** were able to reduce the production of biofilm, with **(*R*)-ASME** resulting in the most effective compound with an EC_50_ of 14 mg/well. The successful application of TFS highlights its usefulness in searching for promising LsrK inhibitor candidates with inhibitor efficacy against biofilm formation.

## 1. Introduction

**Figure 1:**
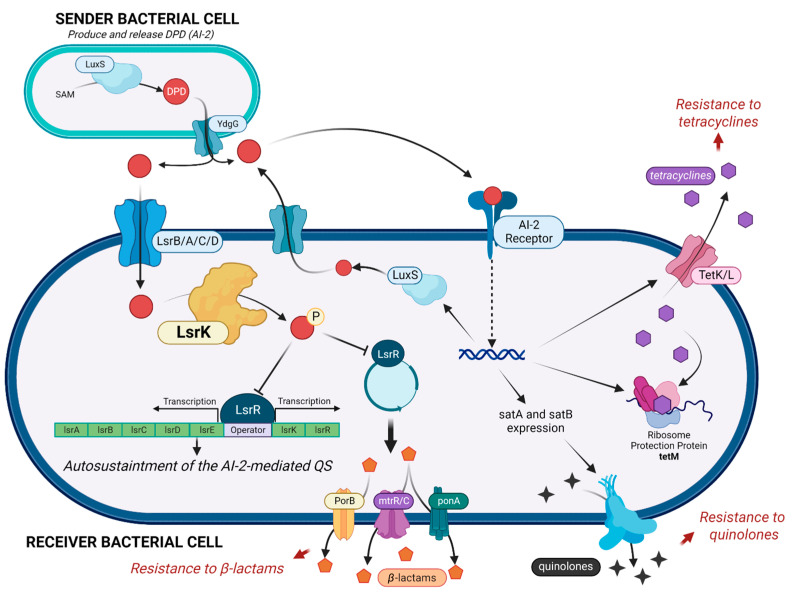
AI-2 mediated cascade, with a focus on the key role of LsrK kinase in controlling the auto-sustaintment of QS and the diverse mechanisms involved in antimicrobial resistance. (Created with BioRender).

Antimicrobial resistance (AMR) and the worldwide increase in superbug infections are recognized by the World Health Organization (WHO) as global concerns for public health and the sustainability of healthcare systems [1]. Superbug bacteria are responsible for about 25% of infections and almost 30% of AMR-related deaths, and they are expected to become the primary cause of death by the year 2050 [1]. To challenge this inauspicious perspective, one of the strategic objectives of the WHO is the research of new pharmaceutical tools and medicines [2]. Overuse, inappropriate prescription, and extensive agricultural use of antibiotics have clearly been exposing bacteria to strong, selective evolutive pressure, leading to the development of protective mechanisms to inactivate, remove, and in general circumvent the toxicity of antibiotics [3]. Conventional antibacterial drugs usually interfere with bacterial cell wall biosynthesis, protein synthesis, or DNA replication, and the search for new antimicrobials is still mainly focused on these strategies, thus exposing these new drugs to the same resistance mechanism [4,5,6]. The search for new targets and innovative antibacterial strategies and weapons has become mandatory. An innovative strategy to deal with AMR is to interfere with biofilm formation and with bacterial quorum sensing (QS). About 80% of all human bacterial infections are complicated by biofilms, within which bacteria have a 1000-fold higher tolerance to antibiotics [6,7]. The QS signaling is the most effective mechanism that bacteria use to communicate and organize in biofilms. QS is mediated by diverse autoinducers. [5] Particularly, AI-2 is produced and sensed by both Gram- and Gram+ bacteria and plays a crucial role in maintaining QS. LsrK is a pivotal key kinase in triggering the QS cascade [8,9,10]. LsrK is responsible for the phosphorylation of Autoinducer-2 (AI-2, the QS messenger most exploited by both Gram-positive and Gram-negative bacteria) to phospho-4,5-Dihydroxy-2,3-pentanedione (P-DPD) [8,11]. This represents the key triggering step in the AI-2-mediated QS cascade (Figure 1). P-DPD binds to the transcriptional repressor LsrR. LsrR dissociates from the promoter region of the *lsrRK* and *lsrACDBFG* operons, inducing the transcription of genes involved in bacterial virulence and in the auto-sustainment of QS [12]. Several studies proved the role of LsrK in bacterial QS [13,14]. LsrK mutants do not activate *lsr* transcription because of the lack of P-DPD with reduced expression of the Lsr transporter and extracellular AI-2 accumulation [13]. Furthermore, when LsrK and ATP were added in the extracellular medium, the phosphorylation of AI-2 outside the cell avoids the internalization of P-DPD due to its negative charge. As a result, a reduction in *lsr* gene expression and attenuation of QS were observed [13].

The multifunctional capabilities of LsrK in the bacterial virulence and AMR make this kinase an attractive candidate for drug development. To date, this target is almost unexplored, and few compounds have been proposed as LsrK inhibitors. However, these pilot studies pave the way for a more complete understanding of the role of the bacterial LsrK kinase inhibition in the autoinducer-2-mediated QS cascade and will allow for the discovery of novel innovative non-antibiotic therapies [8,11]. To date, LsrK inhibitors have been identified by exploiting different indirect strategies (Figure S1) [12]. Briefly, meclofenamic acid (**1**) and its halogenated derivatives **3**–**8** (Figure S1A) were identified via a structure-based virtual screening approach performed on a LsrK homology model developed before the X-ray structures of the kinase were resolved [15]. Compounds **9**–**12** (Figure S1B) were designed by a member of our group by exploiting the structure–activity relationship deciphered around the main backbone of DPD; a spyrocyclohexyl-dioxolane moiety replaced the diol portion essential for LsrK-mediated phosphorylation, whereas the diketo group was embedded in diverse heteroaromatic rings [16]. Lastly, several structural unrelated LsrK inhibitors (Figure S1C) were identified through an automatized target-based HTS, with hapargoside and rosolic acid being active in a cell-based AI-2 QS interference assay [17]. Although the LsrK inhibitory activity for these compounds is reported, no experimental evidence of their binding to the protein was still investigated. Moreover, to complicate the canvas, we still lack experimental evidence of the binding mode of DPD or LsrK inhibitors with the protein, and the putative catalytic binding site has just been postulated thanks to the three LsrK crystallographic structures (PDB ID: 5YA0, 5YA1, and 5YA2) [18] together with the homology comparison with other bacterial kinases, such as glycerol kinase (GK) or xylulose kinase (XK) [18]. As part of our ongoing research on the bacterial Lsrk, in this paper we present the first application of the tryptophan fluorescence spectroscopy (TFS) for studying the ligand–LsrK binding, and we applied this biophysical assay to cherry-pick compounds that potentially target the bacterial kinase from an in-house library of small molecules and secondary metabolites. In addition, the conformational changes of LsrK upon binding with the identified ligands were discussed on the basis of circular dichroism (CD) and the binding of the ligands to the protein was lastly rationalized via molecular docking. These facile methods can provide binding mechanisms between LsrK kinase and potential inhibitors and may facilitate the discovery of novel drugs for fighting AMR through an innovative mechanism of action.

## 2. Results and Discussion

**Figure 2:**
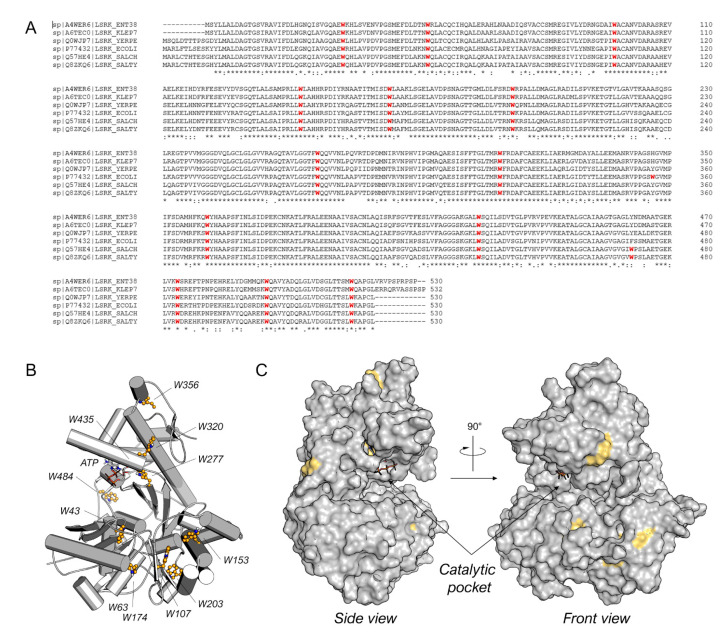
(**A**) Sequence alignment for *Enterobacter* sp. (ENT38), *K*. *pneumonia* (KLEP7), *Y*. *pestis* (YERPE), *E*. *coli* (ECOLI), *S*. *choleraesuis* (SALCH), and *S*. *typhimurium* LsrK. All tryptophan residues are colored in red. Below the sequence alignment is a key denoting conserved amino acid residues (*), conservative substitutions (:), and semi-conservative substitutions (.). The non-conserved tryptophan residues between *E*. *coli* and *S*. *typhimurium* LsrK are highlighted in the red square. (**B**) Tryptophan residue position on the structure of *ec*LsrK (PDB ID: 5YA1). Lsrk is visualized as cartoons in grey. ATP is represented in stick mode. The tryptophan residues are highlighted in ball-and-stick with carbon atom in yellow. (**C**) Visualization of the tryptophan residues exposed on the surface of the protein. The putative catalytic pocket is indicated.

Among the diverse techniques useful for studying the protein–ligand binding, we selected the TFS. Indeed, tryptophan is known to emit intrinsic fluorescence that can be measured via fluorescence spectroscopy [19]. Interactions with binding ligands can change the tryptophan microenvironment, resulting in shifts of the maximum fluorescence peak and variations in intensity [20]. Bacterial LsrK is rich in tryptophan (Figure 2A), with thirteen highly conserved tryptophan residues among diverse bacteria species (e.g., *Enterobacter* sp., *Klebsiella pneumonia*, *Yersinia pestis*, *Escherichia coli*, *Salmonella choleraesuis*, and *Salmonella typhimurium*). Only *E*. *coli* and both *Salmonella* spp. LsrKs possess an additional tryptophan residue in positions 356 and 471, respectively. The location of tryptophan residues on the protein was highlighted on the crystallographic structures of *Escherichia coli* LsrK (*ec*LsrK). As illustrated in Figure 2B,C, four of the thirteen conserved tryptophan residues (Trp43, Trp277, Trp320, and Trp435) are situated close to the catalytic binding pocket, while the remaining are distributed across the protein’s surface. In particular, Trp435 and Trp277 are in close contact with ATP, whereas Trp43 and Trp320 are part of the catalytic cleft, where DPD is supposed to bind.

**Figure 3:**
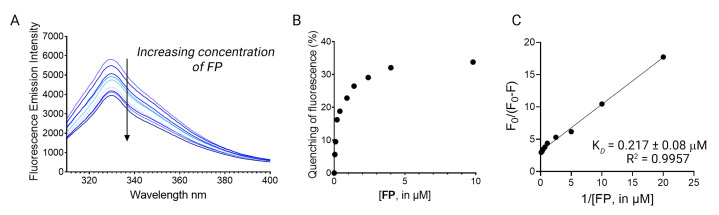
(**A**) The fluorescence quenching spectra of LsrK in the presence of **FP**, [LsrK] = 0.1 μM, [ligand] ranging from 0 to 10 μM, T = 298 K, pH = 7.4, λ_ex_ = 280 nm; the experiments were performed in triplicate; (**B**) % of fluorescence quenching vs. ligand concentration. (**C**) Modified Stern–Volmer plot for the binding of **FP** to LsrK. The points are interpolated using linear regression for the quantification of the K_D_; the values (±SD) are the results of three independent measurements.

The ubiquitous presence of tryptophan residues in the LsrK led us to hypothesize that changes in the intrinsic tryptophan fluorescence, upon ligand binding, might effectively reflect the strength of the LsrK–ligand interactions and the protein conformational change [20]. Based on these premises, we firstly investigated via TFS the secondary metabolite produced by some species of lichen, namely fumarprotocetraric acid (**FP**), which has been isolated by our group within a nature-aided drug discovery (NADD) program. **FP** was used as pilot molecule to investigate its binding with LsrK, since it has been demonstrated as a promising LsrK inhibitor (IC_50_ of 7 μM) [17] and strong antimicrobial agent [21]. Recombinant LsrK was expressed as previously reported [9]. LsrK was titrated by the successive addition of **FP** (from 0.01 nM up to 4 μM). An amount of 1 μL of the appropriate concentration of ligands was progressively added so as to contain the overall dilution of the protein solution below 1%. The fluorescence quenching at 330 nm (the maximum in fluorescence emission) was analyzed. Figure 3A shows the quenching of the fluorescence emission of the Trp residue of *ec*LsrK by the addition of aliquots of **FP** solution. The LsrK fluorescence decreased with the increasing compound concentrations, which suggests that this decrease reflected the interaction of the bacterial kinase with **FP**. The absence of considerable changes in the wavelength of the emission maximum of LsrK is evidence that the presence of **FP** does not exert a great influence on the polarity of the microenvironment of the cavity around the Trp residue. The variation of F_0_/F with the concentration of **FP** (Stern–Volmer plot) was not linear and showed a negative deviation. The negative deviation in the plot may arise by the presence of two populations of fluorophores with different accessibility to the quencher. Indeed, LsrK is a structured protein, thus the tryptophan residues involved in the binding site of the ligand are readily available for quenching, whereas the others not involved in binding will not be quenched (Figure 2). Moreover, some tryptophan residues are buried within the protein, and this contributes overall to a heterogeneous quenching. This condition is reflected in a negative deviation from the linearity of the Stern–Volmer plot [22,23,24,25]. Therefore, the data have been treated using a modified Stern–Volmer equation.

$$\frac{F_{0}}{F_{0}-F}=\frac{1}{f_{a}K_{SV}\left[Q\right]}+\frac{1}{f_{a}}\;$$

where *F*_0_ is the emission fluorescence of LsrK in the absence of ligands, *F* is the emission fluorescence of the protein in the presence of the ligand at the concentration [Q], *f_a_* is the fraction of accessible fluorophore to the ligands, and K_SV_ is the Stern–Volmer constant. The analysis of the data using the modified Stern–Volmer equation is the classical model used in the interpretation of some results of fluorescence quenching in protein solution when the Stern–Volmer plot negatively deviates from linearity, [26,27,28,29,30,31,32,33] since it allows for investigating in detail the quenching mechanism that is occurring between ligands and proteins. Indeed, the linearity of the modified Stern–Volmer plots indicates static quenching (i.e., binding) in the presence of an inaccessible population of fluorophores and the plot allows for the calculation of the dissociation constant (K_D_) and the percentage of fluorophores accessible to the quenchers. *f_a_* is calculated as the reciprocal of the intercept, whereas K_D_ is given by the product of *f_a_* and the slope of the line interpolating the points in the graph [34]. Accordingly, **FP** binds LsrK with static quenching to 26% of accessible Trp residues, and results in a K_D_ of 217 ± 8 nM (Figure 3B).

**Figure 4:**
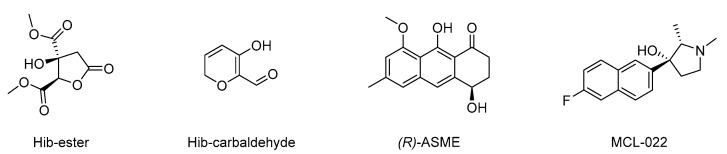
Chemical structure of dimethyl (2*R*,3*S*)-3-hydroxy-5-oxotetrahydrofuran-2,3-dicarboxylate (**Hib-ester**), 5-hydroxy-2H-pyran-6-carbaldehyde (**Hib-carbaldeyde**), *(R)*-(−)-aloesaponol III 8-methyl ether [**(*R*)-ASME**], and (2*S*,3*R*)-3-(6-fluoronaphthalen-2-yl)-1,2-dimethylpyrrolidin-3-ol (**MCL-022**).

Once the suitability of TFS in studying the ligand–LsrK interaction was proven, following the encouraging results obtained with **FP** we screened our in-house compound library for potential ligands that could bind to LsrK. Such chemical libraries are usually assembled from compounds and intermediates produced during other medicinal chemistry investigations performed by research groups in academia and industry. The test’s small molecules were primarily obtained from an in-house collection of compounds (MedChemLab, MCL, Department of Drug Sciences, University of Pavia, Pavia, Italy). The MCL compound library consists of non-commercial small molecules synthesized and assessed during several drug discovery projects performed in the past years by our research group, including several secondary metabolites extracted from the proper plant drugs during nature-aided drug discovery programs [35,36,37,38,39,40,41,42,43,44]. Representative compounds of the MCL library were initially assessed in a low-throughput primary screening against recombinant LsrK via TFS. All the compounds were screened in duplicate at a 100 μM concentration against a 0.1 μM solution of LsrK. The intrinsic fluorescence spectra were then collected in the range of 300–400 nm by keeping the excitation wavelength at 295 nm and the fluorescence emission reduction at 330 nm (the maximum of the emission spectra) was measured. To establish a cutoff point for quenching, we chose **FP** as a reference compound (e.g., reduction in the fluorescence emission > 30% at the tested concentration). Only four compounds (Figure 4) significantly decreased the LsrK fluorescence by more than 30%, namely dimethyl (2*R*,3*S*)-3-hydroxy-5-oxotetrahydrofuran-2,3-dicarboxylate (**Hib-ester**), 5-hydroxy-2H-pyran-6-carbaldehyde (**Hib-carbaldehyde**), (*R*)-(−)-aloesaponol III 8-methyl ether [**(*R*)-ASME**], and (2*S*,3*R*)-3-(6-fluoronaphthalen-2-yl)-1,2-dimethylpyrrolidin-3-ol (**MCL-022**), and they were selected for further characterization. Interestingly, **Hib-ester** and **Hib-carbaldehyde** are two secondary metabolites isolated from the calyces of *Hibiscus sabdariffa*, [35,36,37] whereas **(*R*)-ASME** was isolated from *Eremurus persicus* [38]. Conversely, **MCL-022** is a synthetic compound initially designed as an antinociceptive agent [39,40].

**Figure 5:**
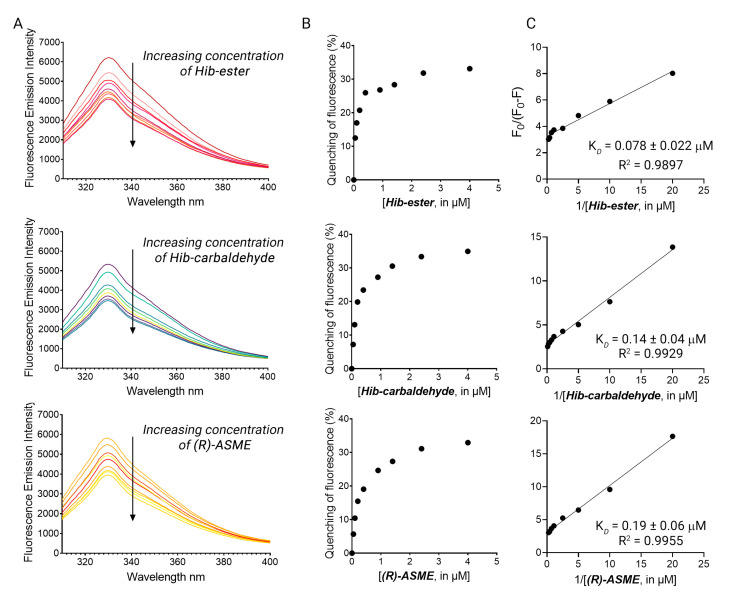
(**A**) The fluorescence quenching spectra of LsrK in the presence of **Hib-ester**, **Hib-carbaldehyde**, and **(*R*)-ASME**, [LsrK] = 0.1 μM, [ligand] ranging from 0 to 4 μM, T = 298 K, pH = 7.4, λ_ex_ = 280 nm; the experiments were performed in triplicate; (**B**) % of fluorescence quenching vs. ligand concentration. (**C**) Modified Stern–Volmer plot for the binding of **Hib-ester**, **Hib-carbaldehyde**, and **(*R*)-ASME** to LsrK. The points are interpolated using linear regression for the quantification of the K_D_; the values (±SD) are the results of three independent measurements.

The four primary hits were validated using dose-response studies to avoid false-positive results, and the K_D_ was determined. The intrinsic fluorescence of the four primary hits alone was recorded first, to confirm the absence of interference between the fluorescence of the compounds with that of the protein. **Hib-ester**, **Hib-carbaldehyde**, and **(*R*)-ASME** did not emit fluorescence at 330 nm when excited at 295 nm. Conversely, **MCL-022** possessed intrinsic fluorescence in the experimental conditions and it was therefore marked as false-positive and discarded. LsrK was titrated by the successive addition of selected compounds as described for **FP**. Figure 5A shows the fluorescence emission spectra in the range 300–400 nm of LsrK in the presence of increasing concentrations of the ligand. **Hib-ester**, **Hib-carbaldehyde**, and **(*R*)-ASME** quenched the intrinsic fluorescence of LsrK in a dose-dependent manner, whereas no quenching of fluorescence emission was detected when only PBS was added to the mixtures of the recombinant protein. Analysis of the tryptophan quenching data using the modified Stern–Volmer equation showed the dependence of F_0_/ΔF on the reciprocal values of the quencher concentration [Q]^−1^ exhibited a good linear relationship, thus confirming that the quenching mechanism between the ligands and LsrK occurred though a static quenching, resulting in K_D_ values of 78, 140, and 190 nM for **Hib-ester**, **Hib-carbaldehyde,** and **(*R*)-ASME,** respectively (Figure 5B,C). To assess whether these compounds might compete with ATP for the binding to LsrK, the binding of ATP for LsrK was studied via TFS, resulting in a K_D_ of 5.07 ± 0.23 µM (Figure S2). The identified secondary metabolites possess a binding affinity 26–63-fold higher that ATP, and therefore they might considerably compete with and displace ATP from its binding site. This suggests a possible ATP-competitive inhibition of the LsrK kinase.

**Figure 6:**
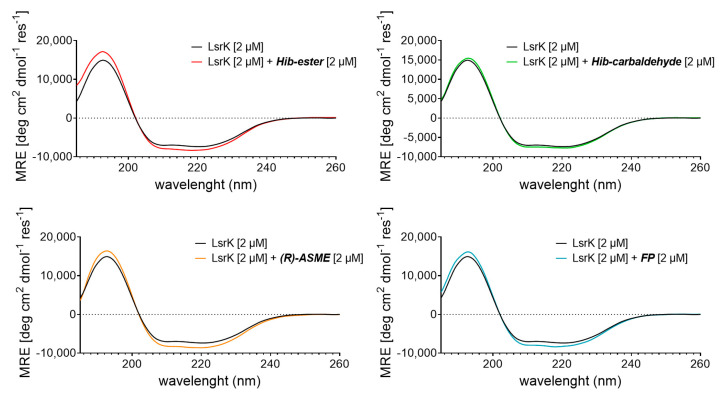
Far-UV CD spectra of LsrK in presence or absence of the indicated compounds. The reported spectra are the average of six scans and corrected for buffer blank.

To characterize in more detail the interaction between the selected natural products and LsrK, we referred to circular dichroism (CD) [45,46]. More specifically, we measured the Far-UV CD spectrum of the bacterial protein in the presence or absence of a saturating concentration of each compound. The results were expressed as the mean residue ellipticity (MRE in deg cm^2^ dmol^−^^1^ res^−^^1^, Equation (S1). Far UV-CD spectra of apo-LsrK at 2 μM revealed a positive band at 195 nm and a negative band with two minimum at 208 nm (n → π*) and 223 nm (π → π*), characteristic of an alpha–helix structure. Deconvolution of the CD spectrum by using BestSel [47] confirmed that the protein is a helix-strand protein and allowed us to estimate the relative content of the secondary structure elements of the protein (Table S1). This result agrees with the crystallographic structure of the protein and the secondary structure content calculated from the available crystallographic structure of the apo-LsrK (PDB ID: 5YA0) [18]. Figure 6 shows the CD spectra of LsrK in the complex with the ligands, in comparison with the spectra of LsrK alone. The presence of each compound modifies the spectrum of the protein, in terms of both an increase in the maximum and a decrease in the minimum, suggesting a change in its conformation as a result of an interaction with the ligands. Briefly, the CD spectra of LsrK upon binding with the four ligands at a molar ratio ligand:LsrK of 1:1 showed an increased ellipticity value at 195 nm and decreased ellipticity at 208 and 223 nm, thereby illustrating that an increase in the α-helical content occurred. Conversely, a slight reduction in the β-sheets and, more importantly, in the turns and the random portions of the protein, was observed. No further variations were observed at a ligand concentration ten times higher than the protein, thus revealing the reaching of saturation binding at 2 μM, as already observed during the TFS studies. This variation was much more marked for **FP**, **Hib-carbaldehyde**, and **(*R*)-ASME** than for **Hib-ester**. This suggests a shift of LsrK towards a more ordered conformation, upon complex formation with the ligands. The variation in the secondary structural components of LsrK upon binding with the four ligands was calculated. When **FP** was present, the contents of the α-helix, β-sheet, β-turn, and random coil structures were changed to 49.5%, 17.6%, 2.6%, and 30.3%, respectively. In the presence of **(*R*)-ASME**, the contents of the α-helix, β-sheet, β-turn, and random coil structures were changed to 47.0%, 16.8%, 0%, and 36.2%, respectively. Similarly, in the presence of **Hib-ester**, the contents of the α-helix, β-sheet, β-turn, and random coil structures were changed to 47.2%, 15.6%, 6.4%, and 30.8%, respectively. Only **Hib-carbaldehyde** induced little variation in the LsrK conformation. Therefore, the CD experimental analysis showed that the interaction of the identified hit compounds with LsrK clearly lead to conformational changes in the protein, thus confirming their binding.

**Figure 7:**
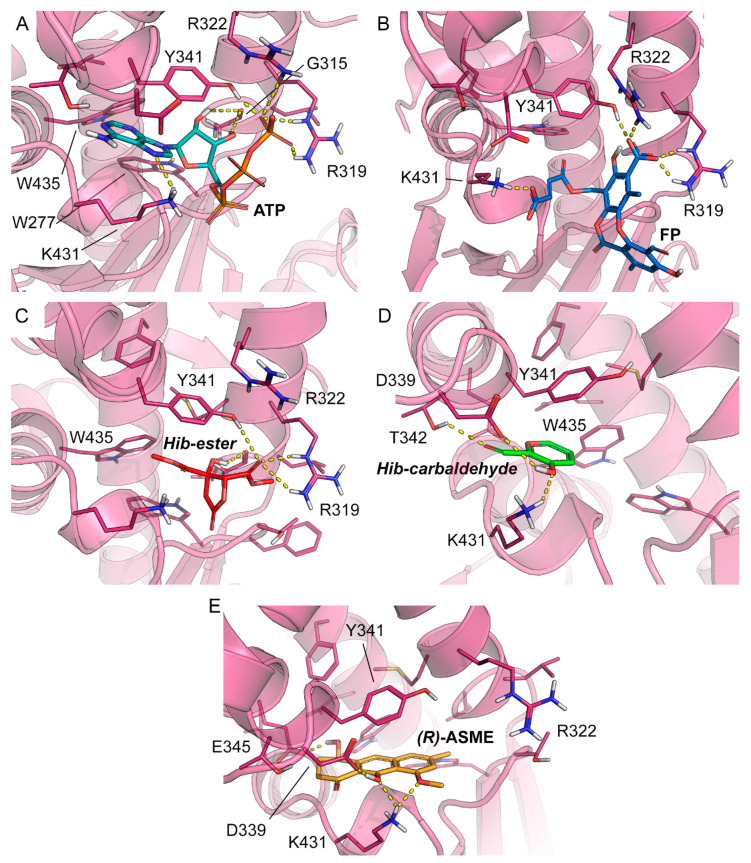
Co-crystallized pose of (**A**) ATP (teal carbon atoms) and the binding poses predicted for (**B**) **FP** (blue carbon atoms), (**C**) **Hib-ester** (red carbon atoms), (**D**) **Hib-carbaldehyde** (green carbon atoms), and (**E**) **(*R*)-ASME** (orange carbon atoms). The reference crystal structure used for the docking calculation (PDB ID: 5YA1) of LsrK is shown in the magenta cartoon; important interacting residues are visualized in sticks representation with magenta carbon atoms. Model atoms except for carbons are color-coded: oxygen (red) and nitrogen (blue). Hydrogen bonds are represented as yellow dotted lines.

To rationalize the spectroscopic results at the molecular level and to identify the likely binding mode of **FP**, **Hib-ester**, **Hib-carbaldehyde**, and **(*R*)-ASME** to LsrK, molecular docking was performed with Glide, which is part of the Schrödinger Platform: Maestro [48,49,50]. More specifically, an X-ray structure of LsrK (in a complex with ATP; PDB ID: 5YA1) was used as receptor for docking [18]. The receptor grid was centered onto the co-crystallized ligand, and ATP and the docking of the four identified secondary metabolites was performed in standard precision mode (“Glide SP”). The docking results indicate that the four investigated small molecules are likely to bind to LsrK in an ATP-competitive manner (Table 1 and Figure 7). With the exception of **Hib-ester**, all of the generated docking poses extend to the deepest part of the ATP binding pocket and are characterized by good protein-ligand surface complementarity. The most convincing and, at the same time, top-ranked docking pose obtained for any of the investigated ligands was obtained for **(*R*)-ASME** (GlideScore—8.184 kcal mol^−^^1^). It is noted that the docking scores (Table 1) do not correlate with the measured K_D_ values. Considering the facts that (i) the highest and lowest measured K_D_ values measured for these compounds differ only by a factor of 2, (ii) the capacity of scoring functions to predict binding free energies is inherently limited, and (iii) the number of data points to test for any correlation is extremely low, this result is well within expectations. Focusing on the binding mode, ATP and all investigated ligands are predicted to form aromatic (or hydrophobic) interactions with Y341. Ligand binding is expected to be sustained by a network of hydrogen bonds (Table 1). Indeed, all investigated ligands are predicted to form at least two hydrogen bonds, primarily with R319, K431, T342, Y342, and/or D339, of which at least one hydrogen bond is known to be formed between ATP and the protein (namely, R319). Overall, the results obtained from docking indicate an ATP-competitive binding of the four compounds of interest.

**Figure 8:**
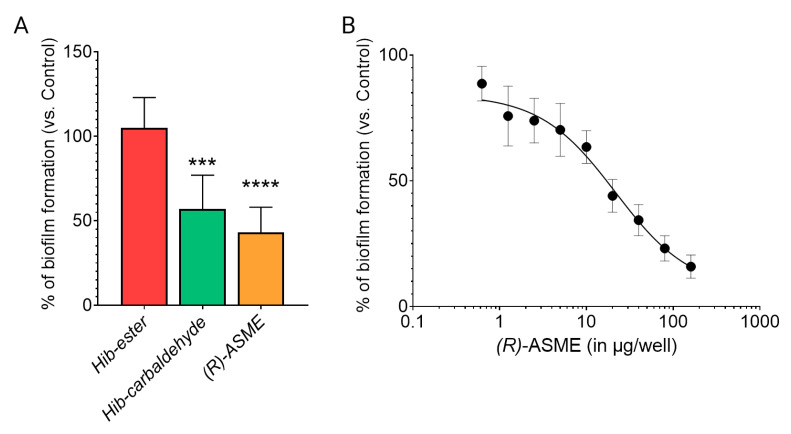
Effect of inhibitors on biofilm formation by staphylococci. (**A**) Staphylococcal cells were incubated with 40 μg/well of each inhibitor at 37 °C for 24 h. Biofilm was stained with crystal violet, and the absorbance was measured at 595 nm. (**B**) Staphylococcal cells were incubated with increasing concentration of ASME at 37 °C for 24 h. Biofilm formation was detected as indicated above. Values are expressed as percentage of the control (no inhibitor added). Data indicate the average of two independent experiments, each performed in quadruplicate. *p* values were determined using a two-tailed, two-sample unequal variance Student’s *t*-test in Prism 4.0 (GraphPad, Prism 4.0, San Diego, CA 92121, USA) with *** *p* < 0.001 and **** *p* < 0.0001 vs. control.

Once the capability in binding LsrK was demonstrated, the efficacy of the three identified compounds in inhibiting the biofilm formation was evaluated on the culture of *Staphylococcus* spp. [7]. **Hib-ester**, **Hib-carbaldehyde**, and **(*R*)-ASME** were initially assessed at 40 μg/well and the biofilm inhibitory activity was reported as percentages of the biofilm produced by the positive control (Figure 8A). Despite the sub-micromolar affinity toward LsrK, **Hib-ester** did not exert any effect on biofilm formation at the tested concentration. Conversely, **Hib-carbaldehyde** and **(*R*)-ASME** caused more than 40% biofilm inhibition at the tested concentration. Clearly, the most active compound was **(*R*)-ASME**, with a biofilm inhibition rate of 60% ± 15 at 40 μg/well, followed by **Hib-carbaldehyde** that exerted a biofilm inhibition rate of 47% ± 22 at the same concentration. Statistical analysis confirmed the significance of the results. **(*R*)-ASME**, which resulted as the most effective compound, was further assessed in a dose-response study resulting in an EC_50_ of 14 ± 4 μg/well, thus indicating its high potential as an effective inhibitor of biofilm formation (Figure 8B). Interestingly, both **Hib-carbaldehyde** and **(*R*)-ASME** did not negatively affected the bacterial viability (measured as planktonic bacterial growth), thus demonstrating that the antibiofilm activity is not correlated with any intrinsic antibacterial activity of the compounds.

## 3. Materials and Methods

### 3.1. Isolation and Purification of Hib-Ester and Hib-Carbaldehyde

The chalices of *Hibiscus sabdariffa* were finely ground using a knife mill, suspended in 80% ethanol, and subjected to microwave radiation. The mixture was cooled to room temperature, filtered, and concentrated under a reduced pressure to obtain the crude extract as a thick reddish oil. The crude was purified via a liquid–liquid extraction and the organic phase was divided into two aliquots and concentrated. The two aliquots were treated in different ways for the isolation of the two metabolites. For the purification of **Hib-ester**, the crude was dissolved in methanol and treated with the PS carbonate anionic resin (Polymer-supported carbonate) to catch all the acidic species contained in the extract. The resin was then treated with 0.1% HCl in methanol, for the recovery of the retained metabolites, thus obtaining a simplified fraction which was further purified via silica gel chromatography to give **Hib-ester** in a 0.22% yield. The yield was calculated on the weight of the vegetable drug used. The second aliquot of crude was subjected to a liquid–liquid extraction using dichloromethane and water. The organic phase was fractionated via chromatography on a silica gel, to isolate **Hib-carbaldehyde** in a 1.1% yield over the weight of the plant drug used.

### 3.2. Isolation and Purification of (R)-ASME

The dried roots of *E. persicus* were extracted with ethanol under microwave irradiation. The suspension was filtered, and the collected solution was concentrated. The ethanolic extract was partitioned between water and dichloromethane. The organic layer was collected, dried, and concentrated. The title compound was purified from the crude via crystallization from acetone.

### 3.3. Isolation and Purification of Fumarprotocetraric Acid

Thalli of *C. foliacea* were finely grinded and extracted with ethyl acetate under mw irradiation. The crude extract was initially treated with methanol to promote the crystallization of usnic acid. The filtrate was concentrated and the fumarprotocetraric acid was recovered via precipitation from the secondary extract via treatment with a mixture of chloroform:ethanol 1:2.

### 3.4. Expression and Purification of LsrK

Culture was grown at 37 °C to an OD_600_ of 0.3, transferred to 22 °C, and grown to an OD_600_ of 0.9. Expression was subsequently induced with 0.1 mM isopropyl 1-thio-β-D-galactopyranoside (IPTG) (Inalco, Milan, Italy) for 9 h. Cells were harvested and resuspended in 25 mM potassium phosphate, pH 7.0, 50 mM NaCl, 10 mM β-mercaptoethanol, 1 mM MgCl_2_, 2.5 µg mL^−1^ DNase, and protease inhibitors and lysed via sonication (70% amplitude, 12 × 30″ on/off, 1′30″ interval between sonication steps). The cell debris was removed via centrifugation and proteins purified from the supernatants via Ni^+2^-affinity chromatography on a HiTrap chelating column (GE Healthcare, Buckinghamshire, UK). Protein purity was assessed via 12.5 % SDS-PAGE and Coomassie Brilliant Blue staining. A bicinchoninic acid protein assay (Pierce, Rockford, IL, USA) was used to measure the concentration of purified proteins.

### 3.5. Fluorescence Binding Studies

Fluorescence quenching experiments were carried out on Jasco spectrofluorometer (FP-6200) using a 10-mm quartz cuvette. The temperature was maintained at 25 ± 0.1 °C using an external thermostatic Peltier device. The stock solution of the ligands was prepared in DMSO and the working solution at the appropriate concentration was prepared in PBS. Protein solution (0.1 μM) was titrated with increasing concentrations of ligand and the fluorescence emission spectrum was recorded in the range of 310–400 nm by keeping the excitation wavelength constant at 295 nm. The excitation and emission slit widths were kept at 5 nm. The final spectra were obtained by subtracting with the corresponding blank. For data analysis, the fluorescence intensity at λ_max_ was plotted against [ligand, μM] and then the modified Stern–Volmer equation (Equation (1)) was used to derive binding parameters viz. binding dissociation constant (*K*_D_).

### 3.6. Circular Dichroism

Far-UV (195–250 nm) CD measurements were performed at 20 °C in a 0.1-cm pathlength quartz cuvette. CD spectra were recorded on a Jasco J-720 spectropolarimeter. Appropriate blanks corresponding to the buffer were subtracted to correct the absorbance or fluorescence background. The results are expressed as the mean residue ellipticity (MRE in deg cm^2^ dmol^−1^ res^−1^), which is defined using the following equation:

(1) $$MRE=\frac{ObservedCD\;\left(mdeg\right)}{C_{p}\times n\times l\times 10}$$

where n is the number of amino acid residues (530 for LsrK), l is the path length of the cell (0.1 cm), and Cp is the molar (M) concentration of the protein. All the spectroscopic measurements were performed in 20 mM phosphate buffer pH 7.4. Six scans were averaged for each spectrum, and the contribution from the buffer was subtracted in each case.

### 3.7. Docking

Docking with Glide in standard precision mode (“SP mode”) was performed on the X-ray structure of LsrK in the complex with ATP (PDB 5YA1). The structure was imported into the Schrödinger Platform for small molecule drug discovery. Next, the X-ray structure was interpreted and processed with the Protein Preparation Wizard (part of the platform) with default settings. As part of this process, the structure was checked for completeness, hydrogens were added, hydrogen bonds optimized, solvent molecules removed, and a restrained minimization of the structure was performed with the OPLS4 force field and the default convergence RMSD tolerance of 0.3 Å. In preparation for docking with Glide, a receptor grid was generated. The enclosing box was centered onto the co-crystallized ligand, ATP. Its size was defined based on the amount of ATP (i.e., the option “dock ligands similar in size to the workspace ligand” was used). The docking algorithm was run with default settings, i.e., the enabled sampling of nitrogen inversions, enabled sampling of ring conformations, and enabled biasing of the sampling of torsions for all predefined functional groups. The hydroxy groups of Y341 and T342 were defined as flexible.

### 3.8. Biofilm Formation

Overnight cultures of staphylococci were diluted 1:200 in Brain Heart Infusion broth (BHI) containing 0.5% glucose. Aliquots (200 μL) of the diluted bacterial suspensions were added to 96-well flat-bottom sterile polystyrene microplates (Costar; Corning, New York, NY, USA) and incubated statically for 24 h at 37 °C in the presence or absence of inhibitors. Biofilms formed on the plates were gently washed twice with phosphate-buffered saline (PBS) (137 mM NaCl, 2.7 mM KCl, 4.3 mM Na_2_HPO_4_ [pH 7.4]) to remove planktonic and loosely adhering bacteria. Adherent cells were fixed with 25% formaldehyde for 10 min, stained with 0.1% crystal violet for 15 min, and after several washings, the wells were air-dried. For a quantitative estimation of the biofilm density, bound crystal violet was solubilized with 10% glacial acetic acid, and the absorbance of the solubilized dye was read at 595 nm in a microplate reader (model 680; Bio-Rad Laboratories, Inc., Hercules, CA, USA).

## 4. Conclusions

To sum up, in this study we apply for the first time TFS as a simple and reliable analytical method for uncovering highly promising LsrK inhibitor candidates. Accordingly, we exploited TFS to identify within an in-house library of synthetic compounds and plant secondary metabolites potential LsrK-binding hit compounds that could provide for new anti-AMR therapeutic drugs. The fluorescence results indicated that the native fluorescence of LsrK is effectively quenched in the presence of the already published LsrK inhibitor **FP** and of the secondary metabolites **Hib-ester**, **Hib-carbaldehyde**, and **(*R*)-ASME**, herein identified via the static quenching mechanism, resulting in a binding affinity for the four compounds to LsrK in the sub-micromolar range. The changes in LsrK conformation observed via CD spectroscopy upon treatment of the protein with the identified hits confirmed the ligand–protein binding and supported the TFS results. Moreover, molecular docking indicated that the binding of the secondary metabolites likely occurs at the ATP binding site, with hydrogen bonds playing a key role in the anchoring of the ligand. Interestingly, **Hib-carbaldehyde** and **(*R*)-ASME** displayed activity against *Staphylococcus* spp. biofilm formation without negatively affecting bacterial viability. **(*R*)-ASME** resulted in the most potent compounds with an EC_50_ of 14 mg/well, indicating its potential as an effective inhibitor of biofilm formation. Thus, this secondary metabolite hold promise as a potential candidate to serve as an effective anti-biofilm drug that can mitigate the risk of bacterial resistance.

## Figures and Tables

**Table 1:** Docking scores and (predicted) interactions of ATP, **FP**, **Hib-ester**, **Hib-carbaldehyde**, and **(*R*)-ASME** with LsrK.

Compound	Residues Involved in the Binding	GlideScore [kcal mol^−1^]
ATP	G315 ^b,d^, R319 ^a,c^, R322 ^a,c^, Y341 ^a,c^, K431 ^a,c^, Y341 ^b,c^	n/a
FP	G315 ^b,d^, R319 ^a,c^, R322 ^a,c^, Y341 ^a,c^, Y341 ^c^	−6.490
Hib-ester	G315 ^b,d^, R319 ^a,c^, Y341 ^b,c^	−4.984
Hib-carbaldehyde	T342 ^a,c^, K341 ^a,c^, Y341 ^b,c^	−6.169
(*R*)-ASME	D339 ^a,c^, K431 ^a,c^, E345 ^a,c^, Y341 ^b,c^	−8.184

^a^ H-bond; ^b^ aromatic/hydrophobic interaction; ^c^ interactions formed with the side chain; ^d^ interactions formed with the backbone. n/a not available.

## Data Availability

The data presented in this study are available on request from the corresponding author.

## Supplementary Materials

The following supporting information can be downloaded at: https://www.mdpi.com/article/10.3390/molecules28062542/s1, Figure S1: Chemical structure, LsrK inhibitory activity and approach exploited for the identification of the LsrK inhibitors reported in literature. Figure S2: % of fluorescence quenching vs. ATP for the binding of ATP to LsrK. Table S1: Percentage of secondary structural components in LsrK.
