# Supplementary material for: Structural Insights into the Ligand–LsrK Kinase Binding Mode: A Step Forward in the Discovery of Novel Antimicrobial Agents
Source: Molecules. 2023 Mar 10;28(6):2542. doi: 10.3390/molecules28062542 (PMC10056567; doi:10.3390/molecules28062542)
Supplement: Supplementary file 1 [file molecules-28-02542-s001.zip › molecules-2259258-supplementary.pdf]

SUPPORTING INFORMATION

Structural insights into the ligand-LsrK kinase binding mode. A step forward in the discovery of novel antimicrobial agents.

Roberta Listro,[1] Giorgio Milli,[1,2] Angelica Pellegrini,[2] Chiara Motta,[2] Valeria Cavalloro,[3] Emanuela Martino,[3] Johannes Kirchmair,[4] Giampiero Pietrocola,[2,*] Daniela Rossi,[1] Pasquale Linciano,[1,*] Simona Collina[1]

[1] Department of Drug Sciences, University of Pavia, viale Taramelli 12, 27100 Pavia, Italy;
[2] Department of Molecular Medicine, Biochemistry unit, University of Pavia, 27100 Pavia, Italy;
[3] Department of Earth and Environmental Sciences, University of Pavia, Via Sant 'Epifanio 14, Pavia, 27100, Italy;
[4] Division of Pharmaceutical Chemistry, Department of Pharmaceutical Sciences, University of Vienna, Vienna, 1090, Austria
* Correspondence: P.L. E-mail: pasquale.linciano@unipv.it. Address: Department of Drug Science, University of Pavia, viale Taramelli 12, 27100, Pavia, Italy. G.P. E-mail: giampiero.pietrocola@unipv.it. Address: Department of Molecular Medicine, Biochemistry unit, University of Pavia, viale Taramelli 3/b, 27100 Pavia, Italy

Table of Content

Figure SI-1. Chemical structure, LsrK inhibitory activity and approach exploited for the identification of the LsrK inhibitors reported in literature. …SI-2
Table SI-1. Percentage of secondary structural components in LsrK. …SI-3

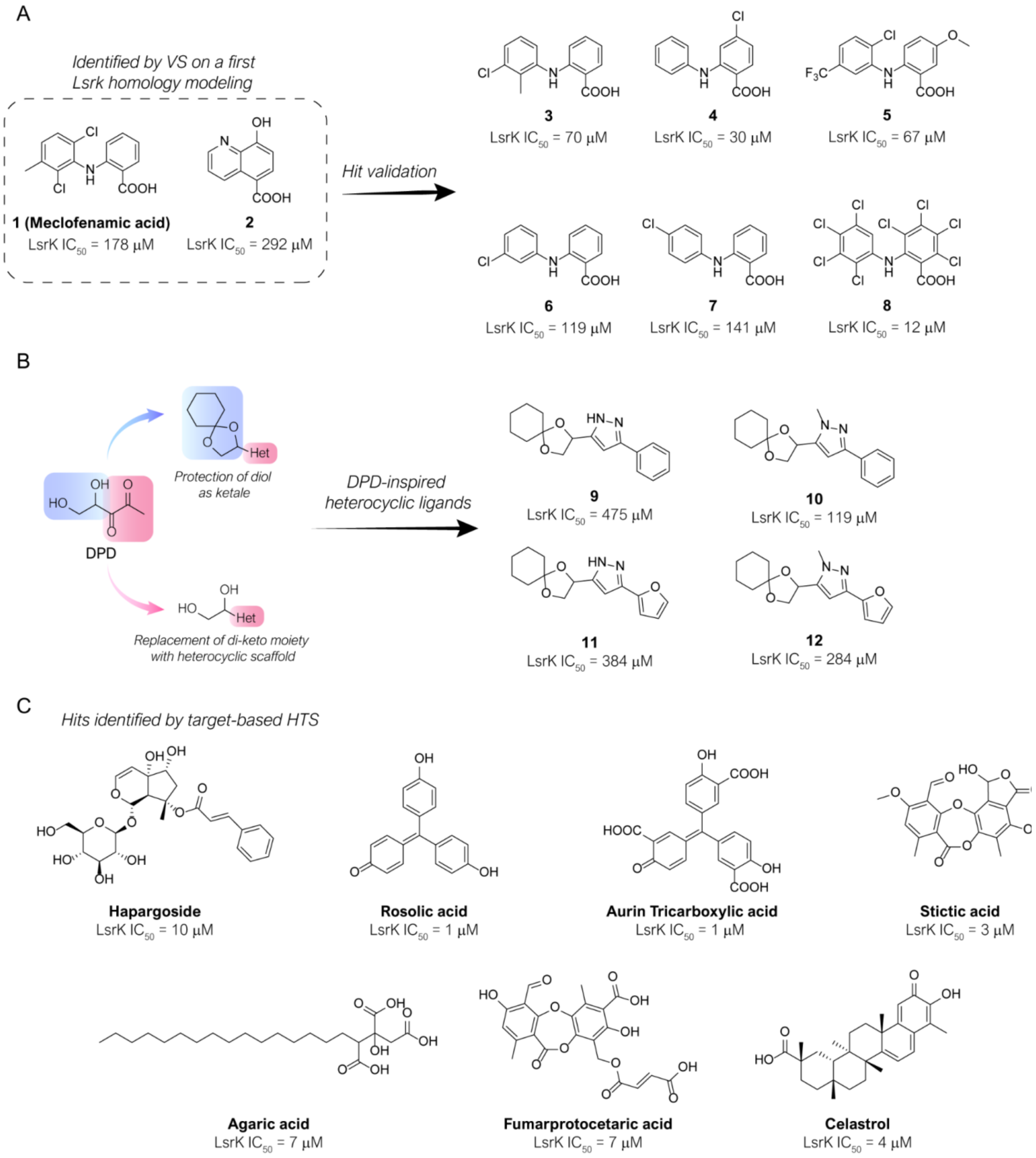

Figure SI-1. Chemical structure, LsrK inhibitory activity and approach exploited for the identification of the LsrK inhibitors reported in literature.

Table SI-1. Percentage of secondary structural components in LsrK.

	**α-Helix**	**β-Strand**	**Turns**	**Other**
from UV-CD spectra[a]	44.4 ± 1.5 %	16.9 ± 2.4 %	3.2 ± 0.3 %	35.7 ± 0.3 %
from apo-LsrK X-ray structure[b]	39.6 %	20.4 %	37.9 %	

a. The values are an average of results obtained using the BestSel deconvolution programs.; b. calculated with the EMBL-EBI PDBsum utility
